# *SOD2* genetic variant associated with treatment-related ototoxicity in cisplatin-treated pediatric medulloblastoma

**DOI:** 10.1002/cam4.516

**Published:** 2015-09-24

**Authors:** Austin L Brown, Philip J Lupo, Mehmet Fatih Okcu, Ching C Lau, Surya Rednam, Michael E Scheurer

**Affiliations:** 1 Section of Hematology-Oncology, Department of Pediatrics, Baylor College of MedicineHouston, Texas; 2 Dan L. Duncan Cancer Center, Baylor College of MedicineHouston, Texas

**Keywords:** Cisplatin, genetics, hearing loss, ototoxicity, platinum chemotherapy, superoxide dismutase

## Abstract

Manganese superoxide dismutase (MnSOD), encoded by the *SOD2* gene, is involved in the detoxification of superoxide anion. Superoxide is likely a source of oxidative stress in the cochlea following treatment with platinum agents and radiation. Therefore, we examined *SOD2* variants in association with ototoxicity among cisplatin-treated childhood medulloblastoma patients. Blood samples were obtained from 71 eligible patients treated for pediatric medulloblastoma at Texas Children’s Cancer Center (1987–2010). Ototoxicity was defined as requiring the use of a hearing aid sometime after the initiation of therapy. DNA was genotyped on the Illumina HumanOmni-1 Quad BeadChip. A linkage disequilibrium (LD)-based single-nucleotide polymorphism (SNP) selection strategy was used to identify a minimal set of informative variants. Associations between SNPs and ototoxicity were assessed using logistic regression. Of the 71 eligible patients, 26 (37%) suffered from cisplatin-related ototoxicity. Study participants were primarily male (73%) and non-Hispanic white (42%). Five *SOD2* variants (rs7855, rs5746151, rs5746136, rs2758331, and rs4880) identified by the LD-based selection strategy were genotyped. After correcting for multiple comparisons, the C-allele of the rs4880 variant was significantly associated with ototoxicity (odds ratio = 3.06, 95% confidence interval: 1.30–7.20) in adjusted models. The rs4880 T > C substitution results in a Val > Ala amino acid change at position 16 of the MnSOD mitochondrial targeting sequence. The Ala variant, which has been associated with increased MnSOD activity, was associated with hearing damage in this study. Platinum-based therapies increase the expression of MnSOD, which may result in an abundance of hydrogen peroxide, a reactive oxygen species. Therefore, oxidative stress may be an important mechanism in therapy-related cochlear damage.

## Introduction

Medulloblastoma is the most common brain malignancy among pediatric populations 1. Current standard of care for medulloblastoma in the United States includes treatment regimens consisting of the platinum-based chemotherapeutic agent cisplatin, which has contributed to long-term survival rates approaching 80% 1. The optimal use of cisplatin chemotherapy, however, is often restricted by the clinical onset of serious dose-limiting side effects. One such side effect is ototoxicity, which can lead to permanent, bilateral hearing loss in up to 70% of patients treated with cisplatin 2–4. Ototoxicity risk has been previously associated with young age at cancer diagnosis, cumulative cisplatin dose, and cranial irradiation 2,5–8. Additionally, a recent genome-wide association study (GWAS) of ototoxicity among pediatric medulloblastoma patients identified common variants in *ACYP2* associated with cisplatin-induced hearing loss 9. However, much work is needed to further characterize the role of inherited genetic variation and individual susceptibility to treatment-related ototoxicity.

While the antineoplastic effect of platinum-containing drugs is attributed to the disruption of cellular mitosis through the formation of DNA crosslinks, evidence suggests that the manifestation of cisplatin ototoxicity involves oxidative damage to the terminally differentiated outer hair cells of the cochlea 10. Under laboratory conditions, exposure to cisplatin increases superoxide anion production, a reactive oxygen species, in the neurosensory epithelium and inner hair cells 11,12. The presence of superoxide anion is necessary to induce apoptosis in HCT116 colon cancer cell lines and has been linked to cochlear toxicity in laboratory animals following treatment with cisplatin 11,13. Oxidative stress can initiate intrinsic, mitochondrial-mediated, apoptotic signaling pathways, indicating cochlear mitochondria may be centrally involved in treatment-related ototoxicity 14. Manganese superoxide dismutase (MnSOD) is a critical component of mitochondrial superoxide anion detoxification, and MnSOD expression has been associated with platinum toxicity in renal epithelial cells 15. Variation in the gene encoding for MnSOD, *SOD2*, has been implicated in noise-induced hearing loss but has not been explored in the context of treatment-related ototoxicity 16. Therefore, we aimed to determine the association between *SOD2* variants and ototoxicity among cisplatin-treated childhood medulloblastoma patients.

## Materials and Methods

A total of 81 pediatric patients diagnosed with medulloblastoma (*n* = 76) or supratentorial primitive neuroectodermal tumors (PNET; *n* = 5), seen at Texas Children’s Cancer Center between 1987 and 2010, who received cisplatin-based chemotherapy, had peripheral blood samples available for genotyping, and had no documented hearing impairment at the time of tumor diagnosis were included in this study. Pediatric PNET patients were included in this analysis if they were treated under medulloblastoma protocols. Of these, 10 patients (one PNET; nine medulloblastoma) were excluded from the analysis due to inadequate medical record documentation of post-therapy hearing status. Informed consent was obtained from the legal guardian of all patients, and the study protocol was reviewed and approved by the institutional review board at Baylor College of Medicine.

Patient characteristics were abstracted from medical records. Information was recorded on a variety of patient demographic and clinical factors, including age at cancer diagnosis, gender, ethnicity, tumor diagnosis, treatment protocol, cumulative cisplatin dose, cranial radiotherapy, surgical procedures, and use of the cytoprotective drug amifostine. A diagnosis of treatment-related ototoxicity was assigned to patients whose medical records indicated that the patient required the assistance of a hearing aid device following the completion of primary therapy.

Genomic DNA was extracted from peripheral blood samples using Qiagen’s (Valencia, CA) QIAamp DNA Mini Kit, following manufacturer instructions. Samples were genotyped on the Illumina HumanOmni-1 Quad BeadChip (San Diego, CA) as part of a separate study. Genetic polymorphisms (SNPs) in close proximity are typically in linkage disequilibrium (LD). A LD-based SNP selection strategy was employed to identify a minimal set of maximally informative *SOD2* single-nucleotide polymorphisms (tagSNPs) 17. The tagSNP approach identifies common variants within a region of a gene such that other known common SNPs within that region are in LD with the selected tagSNP. Genetic regions are typically defined separately by ancestry group because LD structure can vary across populations with different genetic ancestries. *SOD2* tagSNPs were selected for this study utilizing a combined LD threshold of *r*^2^ ≥ 0.8 and a minor allele frequency (MAF) ≥5% from HapMap CEU, YRI, and MEX populations (Table1).

**Table 1:** Selected *SOD2* tagSNPs characteristics among TxCH pediatric medulloblastoma patients (*n* = 71), 1987–2010

tagSNP	Position	Function	Alleles	Global^1^ MAF (%)	TxCH population		
					MAF (%)	Call rate (%)	HWE
rs7855	160100302	3′-UTR	A/G	2.1	2.1	100	0.856
rs5746151	160101320	Intron	G/A	4.2	3.5	100	0.001
rs5746136	160103084	Intron	G/A	30.6	30.3	100	0.783
rs2758331	160105070	Intron	C/A	33.5	42.2	100	0.717
rs4880	160113872	Missense	T/C	41.1	47.2	100	0.570

TxCH, Texas Children’s Hospital; SNP, single-nucleotide polymorphism; MAF, minor allele frequency; HWE, Hardy–Weinberg Equilibrium *P*-value; UTR, untranslated region.

1 Allele frequency from 1000 Genomes phase 1 genotype data release, May 2011.

Statistical analyses were completed in Stata version 13.1 (StataCorp, College Station, TX). A diagnosis of ototoxicity served as the binary outcome of interest. Each tagSNP represented a unique exposure variable; however, tagSNPs with a Hardy–Weinberg equilibrium (HWE) *P* < 0.01 in the patient population were excluded from the analysis. Other variables considered in the analysis included the continuous variables age at diagnosis and cumulative cisplatin dose and categorical variables tumor diagnosis (i.e., medulloblastoma or PNET), gender, ethnicity (i.e., non-Hispanic white, Hispanic, and other), treatment protocol, and amifostine treatment. Craniospinal irradiation dose was also dichotomized at ≥34 Gy, roughly corresponding to the cochlear dose previously associated with detectable hearing impairment in patients with supratentorial tumors while distinguishing between high-risk (36 Gy) and average-risk (23.4 Gy) medulloblastoma treatment regimens 18. Descriptive statistics, calculated as means and ranges for continuous variables or counts and percent of the total for categorical variables, were compared between cases with ototoxicity and control subjects. The association between each tagSNP and ototoxicity was assessed using logistic regression models, assuming a log-additive genetic effect. Clinical variables were considered as potential confounders, and final models adjusted for confounders which were significantly associated with ototoxicity (*P* < 0.05) or the existing literature suggested were clinically relevant. A false discover rate (FDR) corrected *P*-value (*q*-value) was used to account for multiple comparisons, with any tagSNP-associated *q* < 0.05 considered statistically significant.

## Results

Of the 71 eligible patients enrolled in this study, a diagnosis of ototoxicity was satisfied for 26 (37%) of the remaining patients (Table2). Patients were predominantly male (73%), non-Hispanic white (42%), diagnosed with medulloblastoma (94%), and experienced total surgical resection of the tumor (74%). Patients received a mean cumulative cisplatin dose of 328.2 mg/m^2^ (range: 100–720 mg/m^2^); no patient was exposed to other platinum-based agents. Patients with ototoxicity were significantly more likely than control patients to be male (odds ratio [OR] = 4.04, 95% confidence interval [CI]: 1.02–16.03) and have received high doses of craniospinal radiation (OR = 4.46, 95% CI: 1.35–14.71) in multivariable models (results not shown). No other clinical variable was significantly associated with ototoxicity after adjusting for other factors in multivariable models.

**Table 2:** Demographic and clinical characteristics of cisplatin-treated pediatric medulloblastoma patients at Texas Children’s Hospital (*n* = 71), 1987–2010

	Cases^1^ (*n* = 26)	Controls (*n* = 45)	*P*-value
Mean age at diagnosis, year (range)	6.2 (0.7–15.8)	7.5 (1.1–18.0)	0.19
Mean cisplatin, cumulative mg/m^2^ (range)	307 (100–525)	339 (105–720)	0.17
Tumor diagnosis, *n* (%)			0.14
Supratentorial PNET	3 (12)	1 (2)	
Medulloblastoma	23 (88)	44 (98)	
Surgical procedure, *n* (%)^2^			0.06
Biopsy only	1 (4)	0 (0)	
Total resection	15 (60)	37 (82)	
Less than total resection	9 (36)	8 (18)	
Male gender, *n* (%)	22 (85)	30 (67)	0.16
Self-report ethnicity, *n* (%)			0.78
Non-Hispanic white	12 (46)	18 (40)	
Hispanic	9 (35)	15 (33)	
Other	5 (19)	12 (27)	
Treatment protocol, *n* (%)			0.15
SJMB-96/SJMB-03/CCG-9961	17 (65)	35 (78)	
Other with cisplatin	9 (35)	10 (22)	
Craniospinal irradiation ≥34 Gy, *n* (%)	12 (46)	10 (22)	0.06
Amifostine therapy, *n* (%)	8 (31)	20 (44)	0.32

*P*-value from Student’s *t*-test or Fisher’s exact test. PNET, primitive neuroectodermal tumors.

1 Defined as use of a hearing aid device following the initiation of primary therapy.

2 Incomplete information on *n* = 2 participants.

Using a tagSNP approach, we identified five variants on the HumanOmni-1 Quad BeadChip which captured much of the genetic variation in *SOD2* across diverse populations (Table1). Each SNP had a call rate of 100%, though one variant was excluded from the analysis because it significantly deviated from HWE in the patient population (rs5746151). Among the four remaining tagSNPs, the C-allele of rs4880 (OR = 2.16, 95% CI: 1.06–4.38) conferred higher odds of a child developing treatment-related ototoxicity in unadjusted models assuming a log-additive effect (Table3). One tagSNP (rs4880) remained significantly associated with ototoxicity (OR = 3.06, 95% CI: 1.30–7.20) after adjusting for clinical factors and correcting for multiple comparisons (FDR *q* = 0.040). Two additional variants (rs5746136 and rs2758331) were suggestive of a possible association with ototoxicity (FDR *q* < 0.10). Both rs5746136 (*r*^2^ = 0.362) and rs2758331 (*r*^2^ = 0.792) exhibited moderate degrees of LD with the functional rs4880 variant in this patient sample.

**Table 3:** Log-additive association between *SOD2* tagSNPs and ototoxicity among pediatric medulloblastoma patients at Texas Children’s Hospital (*n* = 71), 1987–2010

			Unadjusted model	Adjusted model^2^	
	Case^1^ (*n* = 26)	Control (*n* = 45)	OR (95% CI)	OR (95% CI)	FDR
rs7855			0.86 (0.07–9.97)	0.44 (0.03–6.63)	0.554
AA	25	43			
AG	1	2			
GG	0	0			
rs5746136			2.04 (0.91–4.57)	2.64 (0.98–7.11)	0.072
GG	1	6			
AG	9	9			
AA	16	16			
rs2758331			2.04 (1.01–4.11)	2.59 (1.09–6.15)	0.060
AA	6	8			
AC	16	16			
CC	4	21			
rs4880			2.16 (1.06–4.38)	3.06 (1.30–7.20)	0.040
TT	3	18			
TC	15	18			
CC	8	9			

SNP, single-nucleotide polymorphism; OR, odds ratio; FDR, false discover rate *q*-value.

1 Defined as use of a hearing aid device greater than 1 year removed from the completion of primary therapy.

2 Adjusted for age at diagnosis, gender, ethnic group, cumulative cisplatin dose, and craniospinal irradiation doses ≥34 Gy.

**Figure 1:**
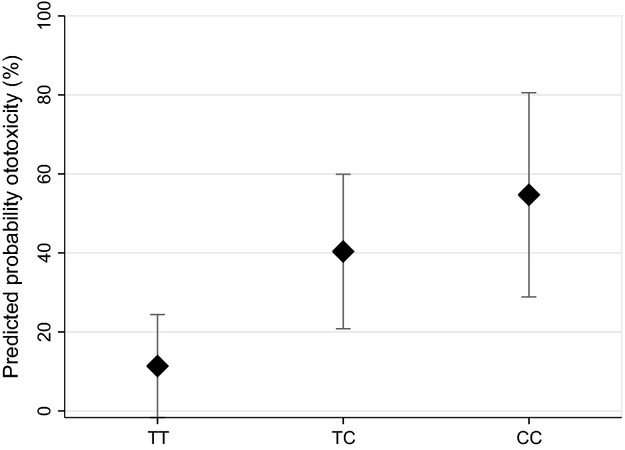
Predicted probability of developing ototoxicity by rs4880 allelic combination among pediatric medulloblastoma patients at Texas Children’s Hospital (*n* = 71), 1987–2010.

In total, 88.5% (23 of 26) of individuals with ototoxicity possessed at least one copy of the rs4880 C-allele (Table3). Similarly, 46% (23 of 50) of C-allele carriers suffered ototoxicity compared to less than 15% (3 of 21) of individuals without a copy of the risk allele. The predicted prevalence of ototoxicity by the allelic combinations of rs4880 is depicted in Figure1, adjusted for relevant clinical variables at the sample mean. The probability of ototoxicity for the TT, TC, and CC genotypes was estimated at 10.8%, 39.4%, and 54.2%, respectively.

## Discussion

Treatment-related ototoxicity is a dose-limiting side effect of platinum chemotherapies, including cisplatin. In this assessment, pediatric medulloblastoma patients who received cisplatin chemotherapy and had the C-allele of *SOD2* rs4880, which has been associated with noise-induced hearing loss 16, had an increased susceptibility to ototoxicity. Nearly 37% of the pediatric patients included in this study experienced clinically relevant ototoxicity requiring the use of hearing aids. Although, the reported prevalence of ototoxicity varies widely with respect to auditory grading criteria, treatment protocol, and other clinical factors, the prevalence of treatment-related hearing loss resulting in the use of hearing aids reported in this study is similar to previously published estimates 19,20. The relationship between ototoxicity and clinical variables observed in this study are largely consistent with other studies 5,7,21–23. Ototoxicity was positively associated with high-dose cranial radiation therapy, male gender, and inversely, though not significantly, related to age at diagnosis (results not shown). Although, amifostine therapy has been associated with a reduced risk of severe cisplatin ototoxicity in average-risk pediatric medulloblastoma patients 24,25, the efficacy of amifostine as an otoprotective agent remains in question. Notably, our results are consistent with a recent meta-analysis that reported decreased ototoxicity in patients receiving amifostine 26. However, as in the meta-analysis, these differences were not statistically significant in our population. This observation is further supported by additional pediatric clinical studies, which did not identify a significant protective effect of amifostine against cisplatin ototoxicity 27–29.

The presence of a cytosine base at rs4880 produces a valine (Val) to alanine (Ala) amino acid substitution at position 16 of MnSOD. This amino acid substitution may alter the structure of the MnSOD mitochondrial targeting sequencing (MTS). While the Val variant likely results in a *β*-sheet MTS structure, the Ala variant may yield a partial *α*-helix structure 30. The results of cellular and in vitro models suggests that the Val variant adversely affects mRNA stability and inhibits efficient mitochondrial import of MnSOD precursors, due to partial arrest of the *β*-sheet structure during transport across the inner mitochondrial membrane 30,31. As a result, the Ala variant is associated with higher levels of the mature MnSOD protein being assembled in the mitochondria and increased MnSOD activity 30,31.

While the mechanism of treatment-related ototoxicity is not well understood, there is some evidence suggesting ototoxicity is a consequence of cisplatin-induced cochlear oxidative stress. The accumulation of reactive oxygen species following cisplatin treatment likely triggers the mitochondrial-mediated apoptotic pathway in the inner ear 14. In particular, cisplatin promotes the production of superoxide, a reactive oxygen species, in the cochlear epithelium and exposure to platinum agents increases MnSOD enzymatic activity, a critical component of mitochondrial superoxide detoxification 11,32. Complete superoxide detoxification requires the sequential actions of MnSOD, which dismutates superoxide into hydrogen peroxide, and downstream enzymes involved in the metabolism of hydrogen peroxide. However, cisplatin exposure disrupts the enzyme equilibrium necessary for complete superoxide anion degradation via down-regulation or inhibition of glutathione, glutathione peroxidase, glutathione *S*-transferase, thioredoxins, and other mitochondrial antioxidant factors 32–36. The combined effects of reduced hydrogen peroxide metabolizing factors and increased MnSOD activity following cisplatin treatment may encourage an overproduction of hydrogen peroxide. Exposure to hydrogen peroxide results in a similar pattern of cellular damage as cisplatin treatment in cochlear epithelial cells, suggesting that hydrogen peroxide availability may contribute to cisplatin ototoxicity 11,37. Hydrogen peroxide production may be particularly high among carriers of the more active Ala variant of MnSOD, explaining the association between carriers of the rs4880 C-allele and ototoxicity susceptibility observed in this study.

Candidate gene studies have explored the relationship between cisplatin ototoxicity and several drug metabolism and oxidative stress genes (e.g., *TPMT*, *COMT*, *GSTP1*, *GSTM1*, *GSTT1*, *ABCC3*) with inconsistent results 4,38–42. A recent GWAS of pediatric embryonal brain tumor patients identified and validated an association between variants in *ACYP2* and cisplatin ototoxicity susceptibility 9. The *ACYP2* gene encodes an acylphosphatase protein (MT-ACP), which can dephosphorylate membrane pump intermediates involved in sodium and calcium homeostasis 43. Although, the role *ACYP2* plays in cisplatin ototoxicity remains undetermined, in vitro and in vivo exposure to hydrogen peroxide can result in the formation of cross-linked MT-ACP disulfide dimers 44. The modified MT-ACP possesses an increased binding affinity for polynucleotides, and nuclear migration of MT-ACP has been observed during apoptosis, suggesting MT-ACP binding and hydrolysis of DNA may be a mediator of apoptosis 43–45. Considered collectively, this evidence supports the hypothesis that variation in the response to oxidative stress may influence susceptibility to cisplatin ototoxicity.

Strengths of the current study include the use of an ethnically diverse population with long-term follow up information. The inclusion of Hispanics and other minority groups, comprising 57% of the study population, increases the generalizability of our findings. Variants included in the analysis were selected using a tagSNP approach incorporating information from HapMap populations (CEU, YRI, and MEX) most representative of our multiethnic study population. The allelic distributions of each tagSNP did not significantly differ by ethnic group in this study (results not shown) and were similar to those reported in other populations 46.

Our study must also be considered in light of certain limitations, including incomplete audiogram and cochlear radiation information, a relatively small sample size and broad window of treatment dates. Instead of using audiogram records, an ototoxicity diagnosis was assigned to patients with medical record documentation of hearing aid use, which has been found to correlate highly with widely accepted audiogram ototoxicity scales (i.e., Chang grade ≥2b) 47. Similarly, craniospinal radiation dose was used to approximate cochlear radiation dose, which was unknown for many of the patients included in this study. Additionally, the *ACYP2* SNPs previously associated with ototoxicity were not genotyped and, therefore, not assessed as part of this study 9. Due to the relatively low frequency of the risk variant (MAF ∼7% according to HapMap population data), we would only expect about five individual to carry the previously reported *ACYP2* risk allele in the current study. Therefore, even if the *ACYP2* SNPs were genotyped, this study was likely underpowered to confirm the reported association with treatment-related ototoxicity. Finally, due to similar treatment regimens and the rarity of pediatric medulloblastoma this study included PNET patients and recruited patients treated over a span of 23 years. Treatment regimens and toxicity monitoring has progressed over the past several decades; therefore, future studies are needed to replicate the results of this study in contemporary populations with comprehensive audiogram and cochlear radiation dose records.

Currently, regular audiometric evaluation is recommended for patients treated with cisplatin and dose modification or termination of cisplatin therapy may be necessary in patients with evidence of significant ototoxicity. Once present, however, treatment-related hearing impairment is typically permanent. Thus, the clinical benefit of ototoxicity monitoring and dose adjustment is limited to reducing the progression of hearing loss. With no effective treatment options, a better understanding of the pathobiology underlying cisplatin hearing loss is needed to reduce the burden of ototoxicity. The etiology of treatment-associated ototoxicity likely involves cochlear exposure to oxidative stress. The results of this and other genetic studies indicate that variation in antioxidant genes influence individual susceptibility to treatment-related ototoxicity. This information could lead to the discovery of novel otoprotective therapeutic targets and help identify patients who may benefit from alternative less ototoxic treatment strategies and chemoprotective interventions.
